# Identification of novel breakpoints for locus- and region-specific translocations in 293 cells by molecular cytogenetics before and after irradiation

**DOI:** 10.1038/s41598-019-47002-0

**Published:** 2019-07-22

**Authors:** Regina L. Binz, Erming Tian, Ratan Sadhukhan, Daohong Zhou, Martin Hauer-Jensen, Rupak Pathak

**Affiliations:** 1 Division of Radiation Health, Department of Pharmaceutical Sciences, College of Pharmacy, University of Arkansas for Medical Sciences, Little Rock, AR USA; 2 Myeloma Center, Winthrop P. Rockefeller Cancer Institute, University of Arkansas for Medical Sciences, Little Rock, AR USA; 3 Department of Pharmacodynamics, College of Pharmacy, Gainesville, FL USA

**Keywords:** Centromeres, Cytogenetics

## Abstract

The human kidney embryonic 293 cell line (293 cells) is extensively used in biomedical and pharmaceutical research. These cells exhibit a number of numerical and structural chromosomal anomalies. However, the breakpoints responsible for these structural chromosomal rearrangements have not been comprehensively characterized. In addition, it is not known whether chromosomes with structural rearrangement are more sensitive to external toxic agents, such as ionizing radiation. We used G-banding, spectral karyotyping (SKY), and locus- and region-specific fluorescence *in situ* hybridization (FISH) probes designed in our lab or obtained from commercial vendor to address this gap. Our G-banding analysis revealed that the chromosome number varies from 66 to 71, with multiple rearrangements and partial additions and deletions. SKY analysis confirmed 3 consistent rearrangements, two simple and one complex in nature. Multicolor FISH analysis identified an array of breakpoints responsible for locus- and region-specific translocations. Finally, SKY analysis revealed that radio-sensitivity of structurally rearranged chromosomes is dependent on radiation dose. These findings will advance our knowledge in 293 cell biology and will enrich the understanding of radiation biology studies.

## Introduction

The human embryonic kidney 293 (HEK-293) cell line is extensively used as a tool for mechanistic studies in mammalian biology and also for propagating viral vectors and generating recombinant proteins in biomedical and pharmaceutical research^1–4^. For many recombinant proteins, posttranslational modifications, such as glycosylation, are essential to the protein’s functional activity, and the 293 system supports these modifications more efficiently than many other mammalian systems widely used in biopharmaceutical industries^5^. Moreover, the 293 cell line and its derivatives are used to: produce lentiviruses^6,7^, study protein–protein interactions^8^, generate homogenously N-glycosylated proteins^9^, and propagate plasmids^10^. Considering its widespread use in various research fields, cytogenetic characterization of 293 cells is critical to understanding how to best utilize this tool for research.

The 293 cell line exhibits a highly complex karyotype, with several numerical abnormalities^1,11^. For unknown reasons, when newly established, the 293 subline had a lower chromosome number than the original parental line. Cytogenetic analyses by various groups demonstrated that the chromosome number varies from 56 to 78^1,12,13^, but about one-third of the cell population carries 64 chromosomes, which is the modal number, according to ATCC (ATCC CRL-1573). These reports demonstrate a lack of consensus on chromosome number in metaphase spreads among various laboratories. Cytogenetic studies also revealed that the copy number of chromosomes X, 1, 6, 11, 17, 18, 20, and 21 varies within the population of 293 cells^12,13^, however, a lack of the Y chromosome is highly consistent in the 293 cells^12,13^, indicating that the cell line originated from a female embryo. The gains or losses of entire chromosomes (i.e., aneuploid chromosome number) are expected result from improper anaphase segregation during the development of the parental cell line. Aneuploidy may occur due to errors in the kinetochore–microtubule attachment, hyper-stable kinetochore–microtubule attachment, presence of extra centrosomes, and disrupted cohesion of sister chromatids at centromeres^14^.

In addition to numerical abnormalities, a series of structural aberrations, including deletions and rearrangements (exchanges or translocations) are commonly observed in 293 cells and its variant forms^12^. Rearrangements can lead to formation of derivative (der) chromosomes, which are generated as a result of more than one structural aberration within a single chromosome or an unbalanced translocation (t) involving two or more chromosomes. Inter-chromosomal rearrangements can be identified accurately by spectral karyotyping (SKY) analysis, which assigns a distinct pseudocolor to each chromosome as a result of hybridization with a combination of five different fluorochromes of specific wavelengths. However, SKY analysis of 293 cells has been reported only once^12^. It showed partial deletion of chromosomes 5 and 8 in 293 cells and daughter cell lines^12^. The same study reported two copies of der(1), which involves an unbalanced translocation (t) between the sub-telomere regions of the long arms (q) of chromosomes 1 and 15 (i.e., der(1)t(1;15) × 2)^12^. Telomere regions that protect the physical ends of linear eukaryotic chromosomes are critical in maintaining the chromosomal stability^15^. A number of other derivative chromosomes with inter-chromosomal translocations also were identified in 293 cells, as follows: der(X)t(X;13), der(6)t(6;19), der(12)t(12;14), der(14)t(14;3), and der(19)t(19;3)^12^. They also reported, in a daughter cell line derived from 293 cells, der(11) with a complex aberration (involving ≥ three breaks in ≥ two chromosomes) that involves translocations of parts of chromosomes 19 and 3 to chromosome 11^12^. This study did not, however, identify breakpoints required for the formation of derivative chromosomes. Moreover, it is not known whether these derivative chromosomes are more susceptible to generate structural rearrangements when exposed to DNA damaging agents, such as ionizing radiation, which is known to induce simple (≤two breaks in two chromosomes) and complex rearrangements^16,17^.

With locus- and/or region-specific fluorescence *in situ* hybridization (FISH) probes designed in our laboratory or procured from a commercial vendor, we demonstrated, for the first time, a number of novel breakpoints responsible for the reported rearrangements in 293 cells. We identified chromosomes with rearrangements by carefully observing the patterns of dark and light bands after G-band staining. SKY analysis was utilized for confirmation for G-banding results. Finally, we determined the effects of radiation on the frequency of structural aberrations, identified the chromosomes involved in the rearrangements, and determined the radio-sensitivity of individual chromosomes with SKY analysis. These findings will augment our knowledge of the 293 karyotype, improve our ability to design mechanistic studies or biomedical and pharmaceutical research with 293 cells, and provide critical information to apply to future radiation biology studies.

## Results

### G-banding shows multiple chromosomes with rearrangements, additions, and deletions

**Figure 1:**
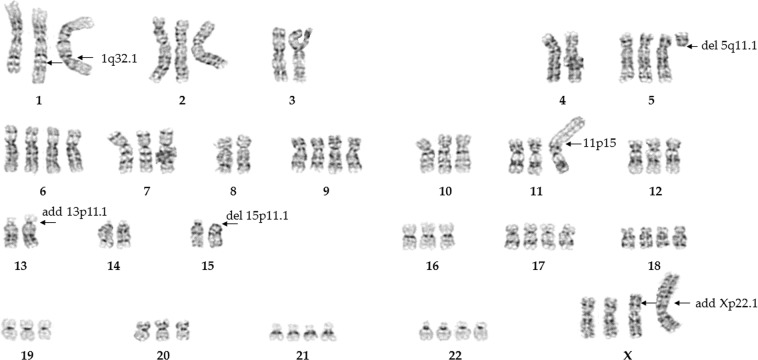
A representative G-band karyogram of a HEK-293 cell showing 71 chromosomes. Arrows indicate rearrangements, additions, and deletions.

We used G-banding to score 10 metaphase spreads of cultured 293 cells. Chromosome number ranged from 66 to 71 (i.e., near triploid, *~3n*). Chromosomes 3, 4, 8, 13, 14, and 15 were consistently diploid in most cases. Chromosome 13 consistently showed an addition of unknown origin distal to 13p11.1. Interestingly, one copy showed complete deletion at 15p11.1. Chromosomes 1, 2, 7, 10, 11, 12, and 16 predominantly were triploid. Notably, 2 out of 3 copies of chromosome 1 had altered banding patterns at approximately 1q32.1, indicating a rearrangement distal to this region. In addition, one copy of chromosome 11 consistently exhibited a large fragment with an unidentifiable banding pattern attached distal to 11p15. We also observed 4 copies of chromosome 17, 21, and X, indicating gain of a whole chromosome. Interestingly, one copy of X chromosome consistently exhibited variation in the banding pattern of distal to Xp22.1; the origin of this altered region is unclear. Further, every cell had a der X chromosome with extra material attached distal to Xp22.3. Chromosome 5 displayed a consistent pattern of partial tetraploidy with a deleted q arm in one copy, indicating a loss of genes exclusively present in the 5q. The remaining chromosomes—6, 9, 18, 19, 20, and 22—were variable in copy number, explaining the variability in overall chromosome counts. Chromosome 6 was the most variable in nature, with 3 to 5 copies. No evidence of chromosome Y was observed. Figure 1 shows a representative G-banded karyogram of a 293 cell. Table 1 and Supplementary Fig. 1 show copy-number variation in each chromosome after G-banding.

**Table 1:** Distribution of copy numbers of individual chromosomes in 293 cells, based on analysis of metaphase spreads after G-banding or SKY painting.

Chromosome number	Copy number of indicated chromosome									
	G-banding (n = 10)					SKY painting (n = 10)				
	1	2	3	4	5	1	2	3	4	5
X				9	1			2	8	
1		1	9					10		
2			10					10		
3	1	9					9	1		
4		10				1	9			
5				9	1				10	
6			3	3	4			1	3	6
7		2	8				1	9		
8		10					9	1		
9		1	5	4				10		
10			9	1				10		
11			10					10		
12		1	9					10		
13	1	9					10			
14		9	1				10			
15		10					10			
16		1	9					10		
17				10				3	7	
18			3	7				1	9	
19			6	4			1	9		
20		4	6				3	6	1	
21				10				1	9	
22			3	7				2	8	

### SKY analysis confirms chromosome-specific translocations

**Figure 2:**
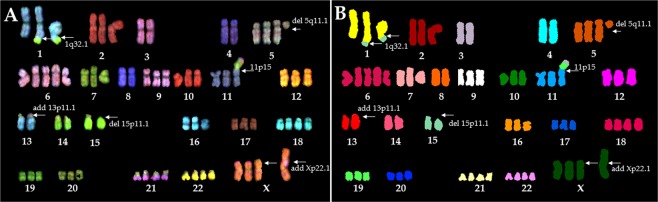
A representative (**A**) spectral and (**B**) classified images of a HEK-293 cell showing 70 chromosomes. Arrows indicate rearrangements, additions, and deletions.

SKY analysis of 10 metaphase spreads allowed us to gain additional information about chromosome-specific rearrangements and investigate any uncertainty resulting from G-banding results. Figure 2 shows a representative karyogram of a 293 cell after SKY “painting”. Table 1 and Supplementary Fig. 2 show copy-number variation for each chromosome after SKY “painting”, which confirms the G-banding data. Specifically, SKY analysis further confirmed the G-banding data that showed chromosome 6 had the greatest variation in copy number (Table 1). SKY painting detected several major inter-chromosomal rearrangements. Distal parts of chromosome 15 were translocated to the 1q32.1 region of two copies of chromosome 1; we did not observe the deleted parts of chromosome 1 in the karyogram, which indicates that these translocations were unbalanced. Chromosomes 19 and 3 were translocated to the region 1 of band 5 onto the short arm (p) chromosome 11 (i.e. 11p15), conferring der(11) with complex rearrangements. The additional segment on der(X) at Xp22.1 was entirely composed of chromosome X, indicating partial gain of the chromosome. No other chromosomes appeared to have rearrangements.

### Multi-color FISH probes reveal locus- and region-specific translocations

#### Presence of two peri-centromere regions with absence of p terminal (pter) in one copy of X chromosome, and a gain of Xq in the derivative X chromosome

**Figure 3:**
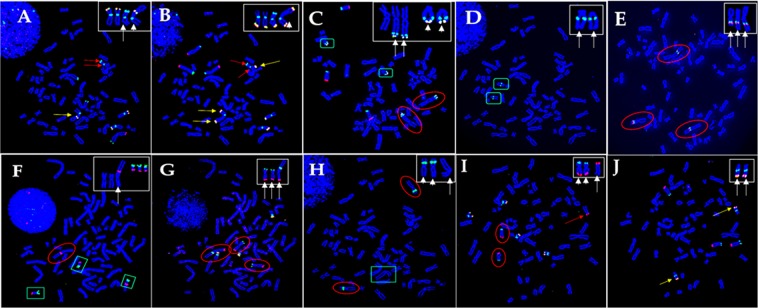
Two sequential hybridizations showing that (**A**) one copy of X chromosome has two peri-centromere regions (aqua) with missing Xpter (arrows), while the derivative X has interstitial Xpter signal (yellow; arrow head/inset), the FISH “cocktail” also contains probes for 1p (green) and 1q (red) and (**B**) re-hybridization confirming the derivative X has Xqter signal (yellow) on either end (arrows and arrow head in the inset), the FISH “cocktail” also containing probes for 2p (green) and 2q (red). (**C**) Translocation of *PML* locus (15q22; aqua) and the sub-telomere region (15qter; yellow) from chromosome 15 (green box; arrow heads/inset) to two copies of chromosome 1 (red circle; arrows/inset). The FISH “cocktail” also contains probes for the sub-telomere regions of chromosome 10 (red and green). (**D**) 15q21 locus is present on chromosome 15 (green box; arrows/inset), not on chromosome 1. (**E**) Confirmation of 1q31 (green) and 1q32 (red) regions are present on three copies of chromosome 1 (red circles; arrows/inset). (**F**) The same metaphase after two sequential hybridizations showing insertion of 19qter into another chromosome (red circle; arrow/inset); (**G**) re-hybridization of the same metaphase confirming the insertion is distal to 11pter as evident from sub-telomere hybridization of p (green) and q (red) arm of three copies chromosome 11 (red circles; arrows/inset); the FISH “cocktail” also contains probes for the sub-telomere (yellow) and centromere (aqua) regions of chromosome 18, and (**H**) absence of 3p21 on the derivative chromosome 11 (green box; arrow/inset), but present on the two copies of chromosome 3 (red circles; arrow heads/inset). (**I**) The same metaphase after two sequential hybridizations showing translocation of 9qter (red) from chromosomes 9 (red circles; arrow heads/inset) to a group D chromosome (arrow); the FISH cocktail also contains positive hybridization for sub-telomere (17qter; yellow) and centromere (aqua) regions of chromosome 17 and (**J**) re-hybridization of the same metaphase confirming the receptor chromosome is chromosome 13 as detected by 13qter (yellow) and 13q14 (aqua) and indicated by arrows in the inset; the FISH “cocktail” also contains probes for 6p (green) and 6q (orange).

We examined telomere and peri-centromere regions of chromosome X in 12 metaphase spreads, after hybridization with a cocktail of labeled probes for 1p, 1q, Xp, and the peri-centromere region of X. Notably, one copy of the X chromosome had two peri-centromere regions with the missing the Xpter (Fig. 3A). The same cell was subsequently hybridized with a mixture of labeled probes for 2p, 2q, Xq, and peri-centromere regions of the X chromosome. The intact der(X) displays postive hybridization for Xqter on both ends, confirming a gain of Xq (Fig. 3B).

#### Translocations of PML locus and sub-telomere regions of chromosome 15 to two copies of chromosome 1 distal to 1q32.1

We investigated the chromosome 15 translocation to 1q by identifying the breakpoint on chromosome 15. We evaluated 10 metaphase spreads after hybridization with a cocktail of labeled probes for 10p, 10q, 15q22 (*PML* locus), and 15qter. The data indicated that the *PML* locus was present in both copies of der chromosome 1 (i.e., der(1)t(1;15)(q32.1;q22) × 2) (Fig. 3C). To further define the breakpoint, we designed and used a custom DNA probe for 15q21, the region just proximal to *PML*. This probe did not hybridize to der(1) × 2, but it did hybridize to chromosomes 15 (Fig. 3D). These findings confirmed that the breakpoints for chromosome 15 were between 15q21 and 15q22. In order to define the breakpoint on der(1) × 2, we designed and used custom DNA probes for 1q31 and 1q32. These probes hybridized both copies of der(1) (Fig. 3E), thus indicating that the breakpoint is distal to 1q32, apparently at 1q41 to 1q44. Importantly, we did not find evidence that these deleted regions were integrated into any other chromosomes in the karyograms of 293 cells.

#### Derivative chromosome 11 includes 19q (but not 19p) and region distal to 3p21

Multi-color FISH probes reveal no evidence of 19p arm in der(11). SKY data indicated that complex rearrangements at 11p15 resulted from translocations of chromosomes 19 and 3. We investigated whether of p and/or q arm of chromosome 19 were present on the der(11). We scored 10 metaphase spreads after hybridization with a cocktail of labeled probes for 19p, 19q, and 19p13. The hybridization pattern clearly demonstrated absence of 19p in the inserted segment (Fig. 3F). Further, we rehybridized the same cell with a cocktail of labeled probes for 11p and 11q, which confirmed the presence of 19q on der(11) (Fig. 3G). Moreover, 11p and 19q were present in “head-to-head” configuration, suggesting telomere association (tas) –tas(11;19)(11p15;19q13.4). To confirm the breakpoint on chromosome 3, we used a custom DNA probe for 3p21. The derivative chromosome 11 did not hybridize with the probe, indicating a breakpoint distal to 3p21 (Fig. 3H).

#### 9q34 translocation to 13p11.1 identified with multi-color FISH probing but not G-banding

Surprisingly, we observed hybridization of 9qter on a D group chromosome (Fig. 3I), which was not detected with analysis of G-banding and SKY analysis. We rehybridized the same cell with a cocktail of labeled probes for 6p, 6q, 13q14, and 13qter. The hybridization patterns confirmed that the receptor chromosome is chromosome 13 (Fig. 3J).

#### SKY analysis reveals frequency and specificity of structural chromosomal aberrations are dependent on radiation dose

**Figure 4:**
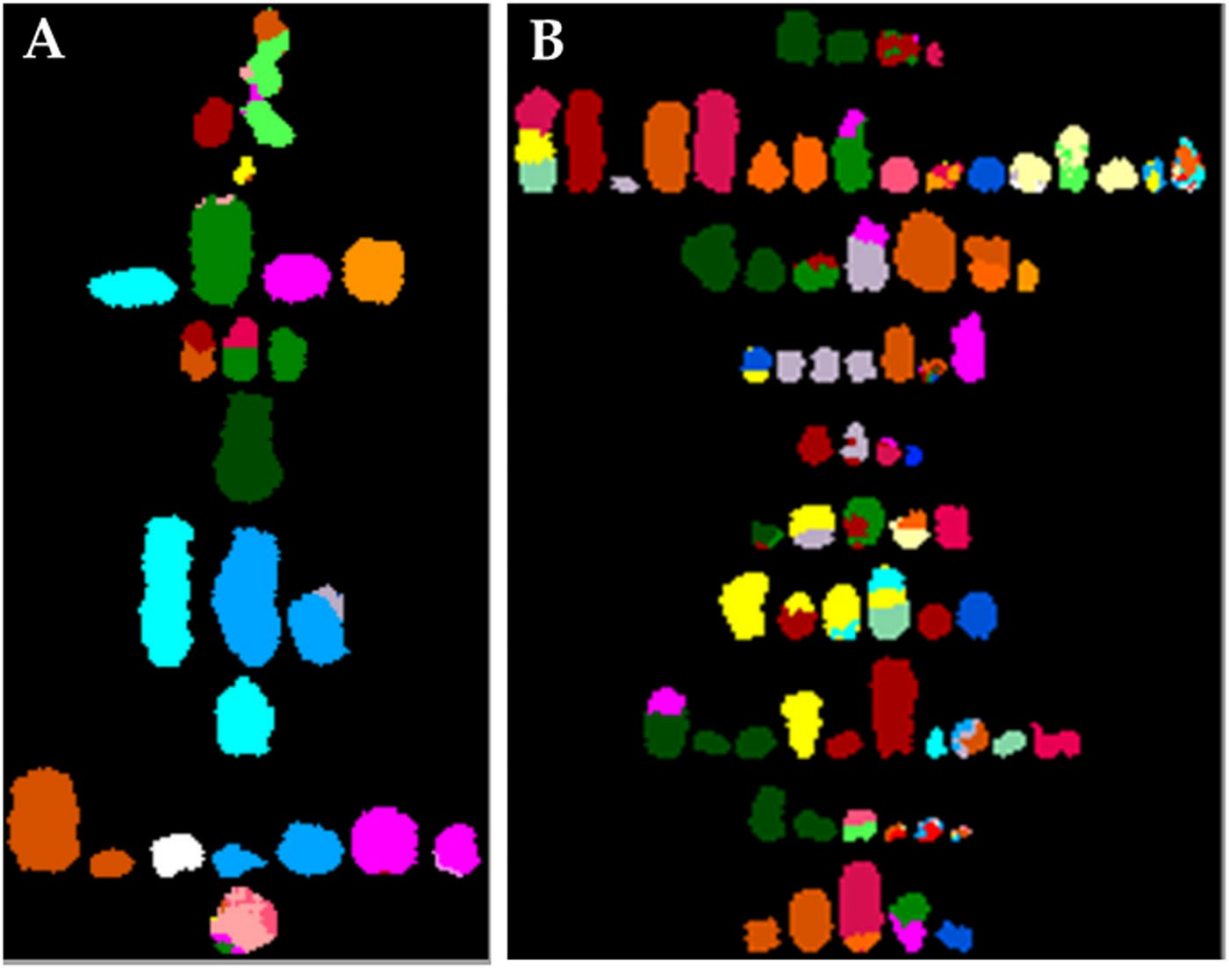
Distribution of acentric fragments in 293 cells by SKY analysis after (**A**) 2 Gy and (**B**) 4 Gy of radiation exposure. A total of 10 metaphase spreads were scored from each treatment group. Each row represents distribution of acentric fragments per metaphase spread.

Finally, we determined the aberration frequency in irradiated 293 cells to evaluate radio-sensitivity of derivative chromosomes by SKY analysis. We exposed cells to 0, 2, and 4 Gy ionizing radiation. We then prepared and scored 10 metaphase spreads from each treatment group 24 h post-irradiation. We observed significant dose-dependent increases in structural chromosomal aberrations (Table 2 and Supplementary Fig. 3) that included dicentric chromosomes, acentric fragments, and ring chromosomes (Table 2). In un-irradiated cells, each spread had 4 color junctions (the intersection of two different chromosomes with distinct color that designate inter-chromosomal exchange), two on der(1) and 2 on der(11). We also observed a dose-dependent increase in acentric fragments generated from individual chromosomes or different chromosomes (Fig. 4). Notably, we did not observe dose-dependent effects on specific numerical or structural aberrations. For example, exposure to 2 Gy resulted in more structural damage to chromosome 11 (4 structural aberrations in 10 spreads), while 4 Gy induced maximum aberrations in X chromosomes (18 structural aberrations in 10 spreads) and in chromosome 1 (18 structural aberrations in 10 spreads). In 4 Gy treatment group, we observed 3 ring chromosomes—two were derivative chromosome 1 and one was chromosome X. Interestingly, in 4 Gy irradiated group, chromosome 11 had the least structural damage, while chromosomes 5 or 12 were involved in majority of dicentric chromosome formation (4 dicentric chromosomes, each involving chromosome 5 or 12, from a total of 11 (8/11) dicentrics observed by SKY).

**Table 2:** Distribution of unstable structural aberration frequencies (±standard errors) detected by SKY analysis in 293 cells after exposure to various doses of ionizing radiation.

Dose (Gy)	Dics	Ace	Rings	Total yield	*p* value
0	0.0 (±0.00)	0.0 (±0.00)	0.0 (±0.00)	0.0 (±0.00)	
2	0.3 (±0.17)	2.4 (±0.48)	0.1 (±0.1)	8.2 (±0.91)	Total yield^*a*^
4	1.1 (±0.33)	7.1 (±0.84)	0.3 (±0.17)	20.3 (±1.42)	Total yield^*a*,*b*^

Dics, dicentric chromosomes; Aces, acentric fragments; ^*a*^statistically significant difference in total yield from 0 Gy dose; ^*b*^statistically significant difference in total yield from 2 Gy dose.

## Discussion

As reported by various groups, 293 cells are aneuploid, with complex karyograms that exhibit 56–78 chromosomes per metaphase spread^11,12,18^; we observed 66–71 chromosomes per metaphase spread, well within the reported range. These reports indicate that 293 cells are near triploid (~*3n*). Similar to previous reports, we found no evidence of chromosome Y and abnormal copy number for chromosomes X, 1, 6, 11, 17, 18, 20, and 21^12^. Our studies demonstrated 3 copies of X chromosome, one der(X) with additional chromatin material, and 4 copies of chromosome 22; these findings corroborate the report by Bylund *et al*. using SKY analysis^12^. We identified two copies of chromosomes 3, 4, 13, 14, and 15, as reported by others^12^. The numerical chromosomal abnormalities we observed in 293 cells match those of previous reports^12^. Our SKY analysis, however, revealed 5 copies of chromosome 6 and 4 copies chromosome 21; while previous study reported only 3 copies of each of these chromosomes^12^. To investigate this discrepancy, SKY analysis from other groups is required; however, such additional data currently is lacking.

This report, consistent with those of others, indicated that 293 cells have multiple structural abnormalities, including gains, losses, and rearrangements of chromosomal segments, which are critical features of the cancer cell karyogram^19^, and drive cancer progression^20^. Notably, injecting 293 cells into nude mice resulted in tumor formation^11^, suggesting that this cell line is a human tumorigenic cellular model. Our G-banding analysis revealed that one copy of chromosome X, 11 and 13 were longer than the other homologous copy of the respective chromosomes, suggesting a partial gain of chromosomal material. We used SKY analysis to determine the origins of the additional chromosomal segments and to identify potential intra- or inter-chromosomal translocations. Results demonstrated that the additional chromosomal material on der(X) was part of X chromosome as the region had the same spectral emission to X chromatin and that the additional chromosomal material on chromosomes 11 and 13 was from different chromosomes. Similar observations were made by Bylund *et al*.^12^, but our studies revealed the previously unreported subtle abnormality of chromosome 13, which involved an unbalanced translocation between chromosomes 13 and 9 (i.e., t(9;13)). This apparent discrepancy could be due to plasticity of structural chromosomal aberrations in 293 cells and scarcity of SKY data for 293 cells in the literature. We also observed consistent deletion of q and p arms in one copy of chromosomes 5 and 15, respectively. A genome sequencing project also revealed deletion of the q arm of chromosome 5^2^.

Others reported one intact copy of chromosome 1 plus two copies of der(1) in 293 cells^12,18^. The der(1) chromosomes have a translocation between chromosomes 1 and 15 (i.e., t(1;15)). Previous G-banding and SKY analysis showed this translocation occurs between band 4 of region 4 on the q arm of chromosome 1 and band 1 of region 2 on the q arm of chromosome 15 (i.e., t(1;15)(q44;q21))^12^. The authors observed this persistent translocation not only in the parental 293 cell but also in the subclones, suggesting clonal selection has no effect on this stable translocation^12^. The translocation also was identified in cytogenetic characterization of 293 cells by ATCC as well as in our G-banding and SKY analysis. However, these staining techniques lack specificity to accurately identify the breakpoints responsible for chromosomal rearrangements.

To gain deeper insights and identify the breakpoints responsible for translocations in 293 cells, we used locus- or region-specific FISH probes. These results uncovered an array of previously unreported structural abnormalities, as follows: one copy of X chromosome devoid of p terminus and containing two peri-centromere regions; der(X) with q terminus on both ends and an interstitial p-terminal region; two copies of der(1), containing the *PML* locus and the distal part of the q terminus of chromosome 15; der(11) devoid of p terminus of chromosome 19; and p-terminal der(13), containing the q terminus of chromosome 9. The significance of these unique rearrangements in modulating cellular functions of 293 cells is yet to be established.

Exposure to ionizing radiation induces various types of cellular damage, but DNA double-strand breaks (DSBs) are the most critical in determining cellular fate. Improper repair of DSBs leads to formation of structural aberrations. Our SKY analysis of irradiated 293 cells showed dose-dependent increases in structural chromosomal aberrations. Similarly, previous SKY analyses demonstrated that structural chromosomal aberrations increase with radiation dose in murine splenic leucocytes and human peripheral blood lymphocytes^21,22^. We did not, however, observe increased frequency of specific structural aberrations or chromosome-specific damage with increasing radiation dose, suggesting no linear correlation between absorbed radiation dose and formation of specific structural aberrations or radio-sensitivity of individual chromosomes. This is consistent with previous work by Mehrotra *et al*.^21^, who observed formation of der(10) involving t(3;10) occurred in splenic lymphocytes after 2 Gy but not 3 Gy exposure. However, 4 Gy data indicates the derivative chromosomes, particularly der(X) and der(1), are more prone to structural damage than other chromosomes. Also consistent with Mehrotra *et al*., we observed numerical chromosomal aberrations in irradiated 293 cells, but no dose-dependent increase in specific numerical aberrations of particular chromosomes^21^. The authors observed that exposure to 2 Gy induces loss of chromosomes 5, 7, and 12 with no addition, while exposure to 3 Gy causes deletion of chromosomes 3, 4, 6, 9, and 10 and gain of chromosomes 9, 18, and 19^21^. These data clearly suggest that radiation-induced numerical and structural chromosomal aberrations are highly complex and are random in nature and that caution is required when using SKY to estimate radiation dose.

**Figure 5:**
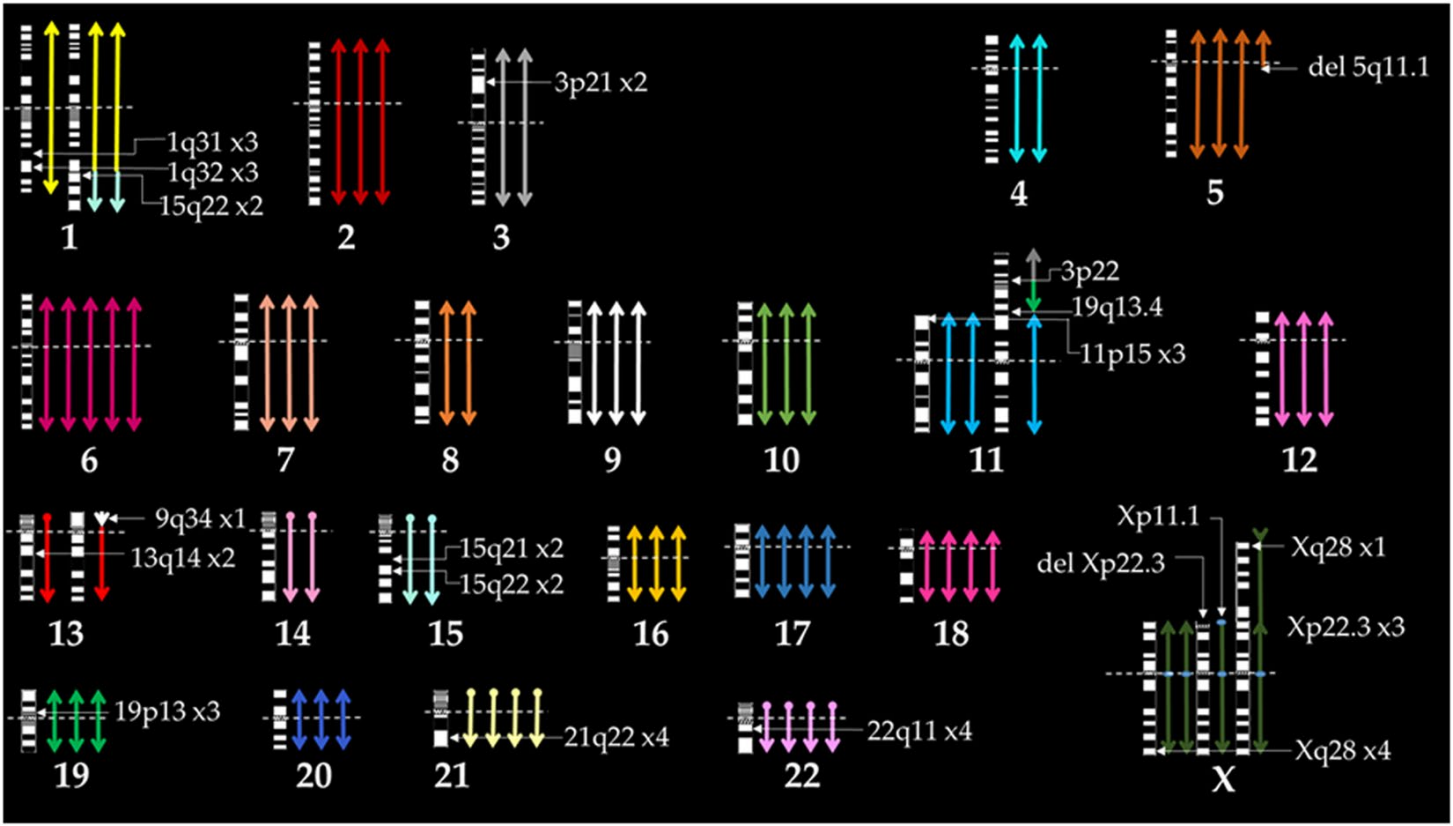
Karyogram of 293 cells showing copy number of individual chromosome, centromere location (as indicated by horizontal dashed line), the break points (as indicated by white arrows), and inter chromosomal rearrangements (by different color junctions) as detected by G-banding, SKY analysis, and locus- and region-specific FISH hybridization.

In conclusion, we used G-banding and SKY analysis to characterize numerical and structural aberrations in 293 cells, and we identified novel breakpoints with locus- and region-specific FISH probes that were designed in-house or obtained from a commercial vendor. On the basis of our molecular cytogenetic analysis, we generated a karyogram of un-irradiated 293 cells (Fig. 5). Based on our novel cytogenetic findings we have created the following composite (cp) karyotype for 293 cell: ~71,XXX, add(X)(p22.1), +der(X)t(X;X)(q13;p22.3), der(1)t(1;15)(q41;q21)x2, −3, −4, +del(5)(q11.1), +6, +6, −8, −11, +der(11)t(3;19;11)(3pter->3p22::19q?->19qter::11pter->11qter), −13, der(13)t(9;13)(q34;p11.1), −14, +15,del(15)p11.1, +17, +18, +21, +22[cp10]. Our SKY analysis data demonstrated radiation increases in structural chromosomal aberrations in dose-dependent manner; however, no specific aberration increased with increasing radiation dose, suggesting lack of dose-dependence for any particular structural aberration. However, one should take into consideration that our small sample size could be the reason for not achieving a correlation of dose-dependent increase in any chromosome-specific aberration. The identification of novel breakpoints will allow us to better understand how the translocations impact genes and phenotypes of 293 cells. This knowledge will also enable us to unambiguously identify the potentially affected/disrupted genes or chromosomal regions. For example, whether gain of Xq influences tumorigenic properties, or amplification and translocation of 15q22 locus containing the *PML* gene to chromosome 1 alters proliferation of 293 cells. These novel findings will enrich our knowledge of 293 cell biology.

## Materials and Methods

### Cell culture

HEK293 cells were purchased from ATCC (Manassas, VA) and cultured in Dulbecco’s Modified Eagle Medium (DMEM; Gibco, Grand Island, NY) supplemented with 10% fetal bovine serum (Gibco). Cells were maintained in a humidified incubator with 5% CO_2_ at 37 °C. Cells were subcultured every 3 days after dissociation with a brief trypsin (Gibco) treatment. Experiments were performed in passage number 15.

### Irradiation

Cells were grown in T25 flasks (Corning, Corning, NY) and were exposed to 2 Gy and 4 Gy ionizing radiation with a Shepherd Mark I ^137^Cs irradiator (model 25, J. L. Shepherd & Associates, San Fernando, CA). Sham irradiated (0 Gy) cells served as a control. Flasks were placed on a turntable rotating at 6 rpm to ensure uniform dose distribution. The average dose rate was 1.01 Gy/min and was corrected for decay each day. At least once a year, dosimetry of the irradiator is performed with gafchromic film and alanine tablets, analyzed by the National Institute of Standards and Technology (NIST), and with ion chambers calibrated yearly in a NIST-traceable laboratory (University of Wisconsin). Dosimetry is overseen by Dr. Narayanasamy, board-certified medical physicist at UAMS.

### Chromosome preparation

Metaphase chromosomes were prepared as described elsewhere^23,24^. Cells were treated with KaryoMAX colcemid (Gibco) at a final concentration of 75 ng/ml for 20 min to arrest cells in metaphase. Cells then were washed with PBS without Ca^++^ and Mg^++^ (Gibco), followed by trypsin treatment to dislodge adherent cells, and centrifuged at 1000 rpm at room temperature. The supernatant was removed, and the cell pellet was gently resuspended in prewarmed hypotonic solution (75 mM KCl; Gibco). Cells then were incubated in 37 °C water bath for 15 min. After hypotonic treatment, cells were fixed by gently adding 500 μl of acetomethanol (3:1, methanol: acetic acid) with gentle and thorough agitation in order to ease cells into fixation. After centrifugation for 10 minutes at 1000 rpm, the cell pellet was gently re-suspended in 2 mL of supernatant and fixative was added in a dropwise manner with constant agitation for a total of 4 mL. An additional 4 mL of fixative was added and the cells were allowed to rest at room temperature for 20 min. After two additional washes with fixative solution, cells were dropped onto pre-cleaned glass slides at 20–25 °C with 42–45% humidity to obtain optimum spreading of metaphase chromosomes.

### G-banding

Trypsin-Giemsa staining was used to prepare G-banded chromosomes^25^. Slides were baked overnight at 66 °C and treated with 0.025% trypsin for 1 min, gently rinsed with Tyrode’s buffer (Sigma, St. Louis, MO), and stained with Giemsa (Sigma) for 5 min. Karyotype integrity of 293 cells was determined after G-banding, according to the International System for Human Cytogenetic Nomenclature. For karyotyping, images were acquired with Zeiss Imazer.Z2 microscope equipped with GenASIs Case Data Manager system, version 7.2.2.40970. At least 10 well-spread randomly selected metaphase spreads were scored.

### Spectral karyotyping (SKY)

We used SKY^23^ to accurately identify inter-chromosomal structural aberrations as detected by altered banding patterns after G-banding. We used the SKY kit from Applied Spectral Imaging (ASI, Carlsbad, CA) according to the manufacturer’s protocol; the probe mixture and hybridization reagents were prepared as recommended. Briefly, slides were aged at room temperature overnight. Just before hybridization, slides were soaked in 2× SSC (Sigma) at room temperature for 5 min and dehydrated in 70%, 80%, and 100% ethanol for 2 min each. Slides were denatured in pre-warmed denature solution (70% formamide [Millipore, Temecula, CA] in 2× SSC) at 71 °C for 1 min and immediately placed into a cold ethanol series (70%, 85%, 100%; 2 min each). SKY probes were denatured in a water bath at 80–81 °C for 7 min. After denaturation, 10 μl of denatured probe mixture was applied to the target area of the slide, covered with a 22 × 22-mm coverslip, and sealed with rubber cement. Slides were placed in a humidified light-protected box and allowed to hybridize at 37 °C for 48 h. Hybridized slides then were washed with formamide buffer (50% formamide in 2× SSC) pre-warmed to 45 °C three times, followed by two washes in 1× SSC for 5 min each. Slides were quickly dipped in distilled water and dried, 60 μl of blocking reagent was applied to the target area and protected with a plastic coverslip. Slides were incubated for 30 min at 37 °C. Cy5 and Cy5.5 antibody staining reagent (ASI) was reconstituted with filtered 4× SSC. The coverslip was carefully removed, 60 μl Cy5 antibody solution was applied to the target area and protected with a clean plastic coverslip. Slides were incubated for 1 h at 37 °C. Slides were washed three times with pre-warmed 4× SSC containing 0.1% Tween-20 for 5 min each at 45 °C. Each slide was briefly dipped into distilled water to remove detergent residue. Cy5.5 antibody solution was applied to the target area and protected with a plastic coverslip. The slides were again incubated for 1 h at 3 7 °C. The 4× SSC/0.1% Tween wash was repeated, as was the brief dip into distilled water. DAPI counterstain was immediately applied, and a clean glass coverslip was placed over the target area. Image acquisition for SKY was performed with an SD200 Spectracube (ASI) mounted on a Zeiss Imager.Z2 microscope. DAPI images were captured and then inverted and enhanced with SKY View software to produce G-band–like patterns on the chromosomes. SKY images were captured under 63× magnification. The visualization of all human chromosomes in different colors is achieved by spectral imaging. Spectral imaging combines fluorescence microscopy, CCD-imaging and Fourier spectroscopy to visualize simultaneously the entire spectrum at all image points.

### FISH technique and designing of custom FISH probes

To further delineate the structural aberrations observed with SKY technique, we used locus- and centromere and/or telomere-specific FISH probes with the aim of elucidating specific chromosomal breakpoints. FISH probes were designed in our lab, as described elsewhere^26^ or obtained from Vysis Inc./Abbott Molecular Laboratories (Abbott Park, IL). We designed the following region-specific probes in our laboratory: Xp11, 1q31 (*ASPM*), 1q22, 3p21, 15q21, and 19p13. Briefly, custom probes were made from Bacterial Artificial Chromosome (BAC) or P1 Artificial Chromosome (PAC) clones containing the desired genomic region in our lab as described elsewhere. BAC/PAC DNA was purified using NucleoBond Plasmid Kit (Takara Bio USA, Inc., formerly known as Clontech Laboratories, Inc., Mountain View, CA). The FISH probes were labeled in a nick translation reaction to incorporate Spectrum-red/green-dUTP (Vysis Inc./Abbott Molecular Laboratories) into DNA templates. In brief, 1 µg of BAC/PAC DNA was mixed with 10 µM of Spectrum-dUTP, 5 µM of dTTP, and 15 µM each of dATP, dCTP, and dGTP in nick translation buffer and enzymes (Vysis Inc./Abbott Molecular Laboratories). The reaction was carried out at 15 °C for 2 hours. Human Cot-1 DNA (Invitrogen Corp., Carlsbad, CA) was also added to block repetitive DNA sequences followed by ethanol precipitation, then suspended in 100 µl of DNA *in situ* hybridization solution (DAKO Co., Carpinteria, CA).

We also used ToTelVysion probes (Vysis Inc./Abbott Molecular Laboratories), which are 15-probe cocktails, to demark the sub-telomere regions of all human chromosomes. All FISH probes were used according to the previously described protocol^23^. Briefly, just before hybridization, slides were soaked in 2× SSC (Sigma) at room temperature for 5 min and dehydrated in 70%, 80%, and 100% ethanol for 2 min each. Slides were denatured in pre-warmed denature solution (70% formamide [Millipore, Temecula, CA] in 2× SSC) at 71 °C for 1 min and immediately placed into a cold ethanol series (70%, 85%, 100%; 2 min each). FISH probes also were denatured in a water bath heated to 75–77 v °C for 5 min. After denaturation, 10 μl of denatured probe mixture was applied to the target area of the slide and protected with a 22 × 22 mm coverslip, which was sealed with rubber cement. Slides were placed in a humidified light-protected box and allowed to hybridize overnight at 37 °C. Hybridized slides then were washed with formamide solution (50% formamide in 2× SSC) pre-warmed to 45 °C three times, followed by two washes in 2× SSC for 5 min each. Slides were quickly dipped in distilled water air dried, and immediately counterstained with DAPI. Cells were photographed and analyzed with GenASIs software (ASI).

### Aberration scoring technique

We scored various types of numerical and structural aberrations with conventional G-banding analysis and using molecular cytogenetic techniques, such as, FISH and SKY at 63× magnification, as described elsewhere^27,28^. At least 10 metaphase spreads were scored for each staining technique. Loss or gain of chromosomal material is considered deletion or addition, respectively. A chromosomal aberration was considered complex if it involved three or more breaks in two or more chromosomes. Deletions, insertions, and terminal and reciprocal translocations were counted as simple aberrations. A rearrangement was counted as simple when it is consisted of maximum of two breaks in two chromosomes. The number of color-junctions per cell is a simple parameter representing the frequency of improper rejoining of chromosomes, and the number of excess painted fragments represents acentric fragments.

### Statistical analysis

Statistical analysis was performed with online GraphPad Prism software. Aberration frequency was calculated by dividing the number of aberrations observed by the total number of metaphase spreads scored; we scored 10 metaphase spreads from each treatment group. Standard errors for the frequencies were calculated by $\sqrt{a}/A$, where *a* is the number under consideration and *A* is the total number of metaphase spreads analyzed, as described elsewhere^28,29^. Differences in induction of various types of aberrations between two groups were calculated with an unpaired *t*-test. Differences in aberration induction between two groups were considered statistically significant when *p* was less than 0.05.

## Supplementary information

Supplementary figures
