# Supplementary material for: Identification of novel breakpoints for locus- and region-specific translocations in 293 cells by molecular cytogenetics before and after irradiation
Source: Sci Rep. 2019 Jul 22;9:10554. doi: 10.1038/s41598-019-47002-0 (PMC6646394; doi:10.1038/s41598-019-47002-0)
Supplement: Supplementary file 1 — Supplementary figures [file 41598_2019_47002_MOESM1_ESM.pdf]

Title: Identification of novel breakpoints for locus- and region-specific translocations in 293 cells by molecular cytogenetics before and after irradiation

Regina L. Binz[1], Erming Tian[2], Ratan Sadhukhan[1], Daohong Zhou[1, 3], Martin Hauer-Jensen[1], Rupak Pathak[1*]

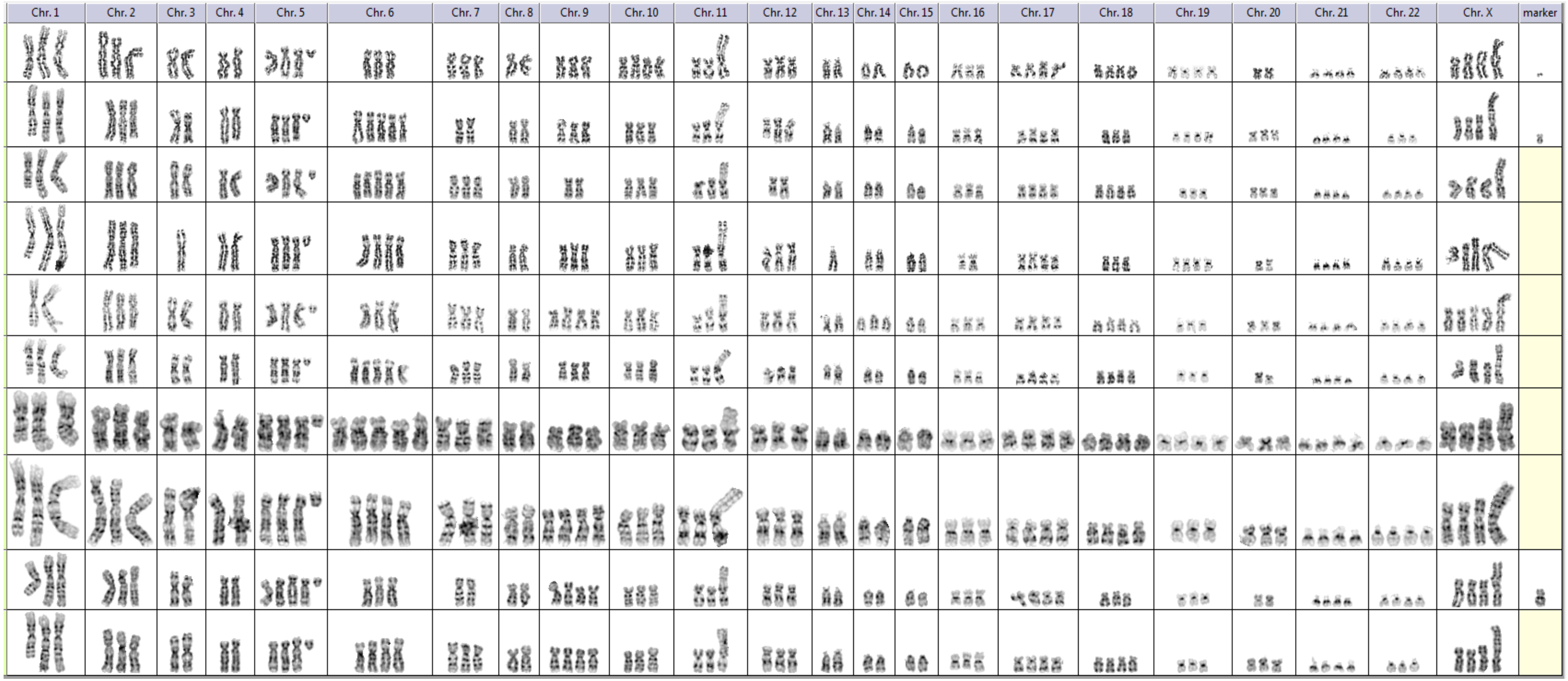

Supplemental figure 1. Distribution of copy number of each individual chromosome by G-banding in 10 metaphase spreads.

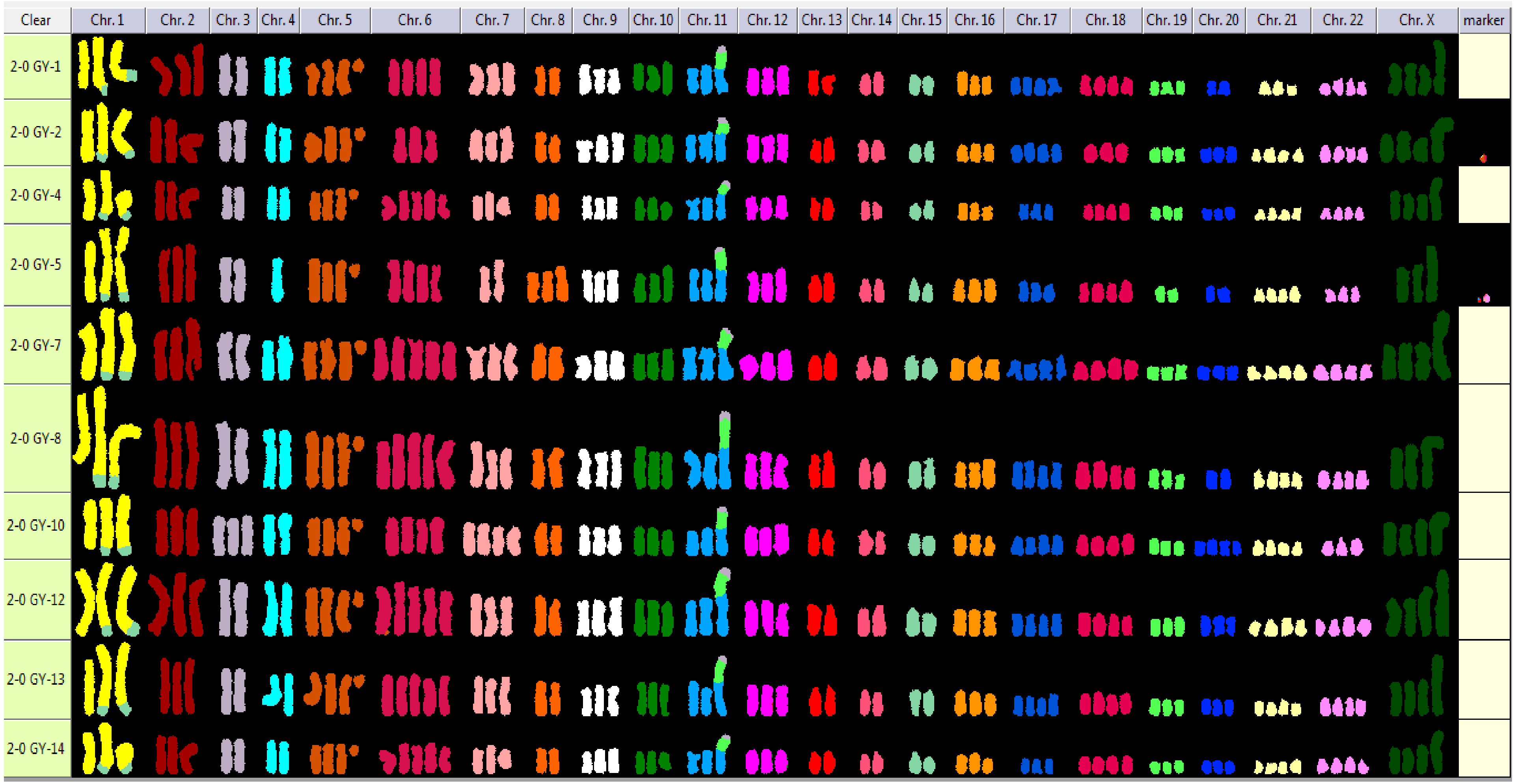

Supplemental figure 2. Distribution of copy number of each individual chromosome by SKY analysis in 10 metaphase spreads.

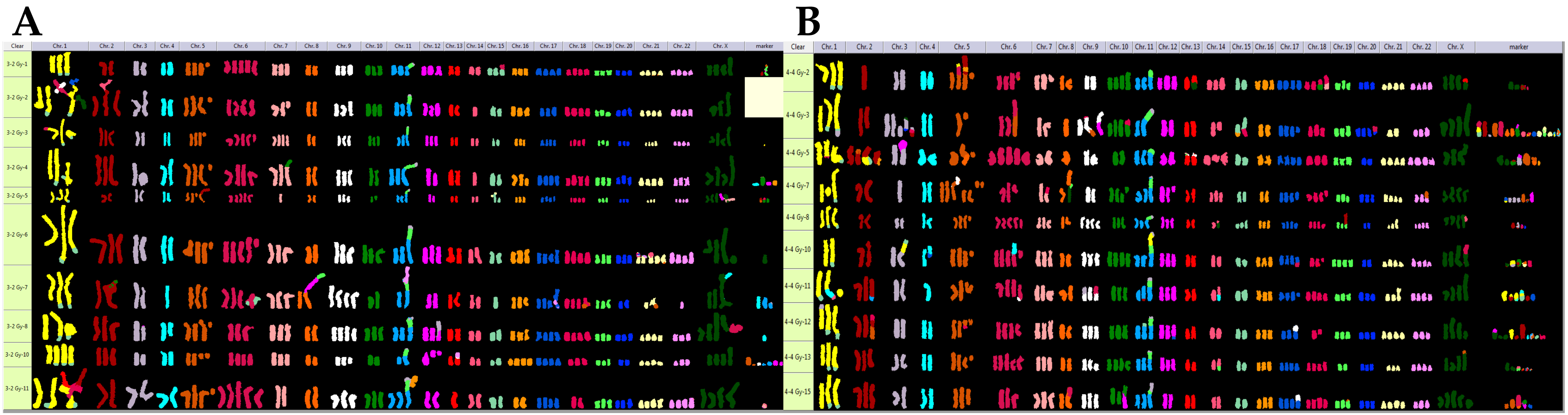

Supplemental figure 3. Distribution of numerical and structural chromosomal aberrations in 293 cells by SKY analysis in 10 metaphase spreads after exposure to (A) 2 Gy and (B) 4 Gy of ionizing radiation. The last column showing the radiation-induced acentric fragments in each metaphase spread.
